# Pathogen-Mediated Proteolysis of the Cell Death Regulator RIPK1 and the Host Defense Modulator RIPK2 in Human Aortic Endothelial Cells

**DOI:** 10.1371/journal.ppat.1002723

**Published:** 2012-06-07

**Authors:** Andrés G. Madrigal, Kenneth Barth, George Papadopoulos, Caroline Attardo Genco

**Affiliations:** Department of Medicine, Section of Infectious Diseases, Boston University School of Medicine, Boston, Massachusetts, United States of America; Tufts University School of Medicine, United States of America

## Abstract

*Porphyromonas gingivalis* is the primary etiologic agent of periodontal disease that is associated with other human chronic inflammatory diseases, including atherosclerosis. The ability of *P. gingivalis* to invade and persist within human aortic endothelial cells (HAEC) has been postulated to contribute to a low to moderate chronic state of inflammation, although how this is specifically achieved has not been well defined. In this study, we demonstrate that *P. gingivalis* infection of HAEC resulted in the rapid cleavage of receptor interacting protein 1 (RIPK1), a mediator of tumor necrosis factor (TNF) receptor-1 (TNF-R1)-induced cell activation or death, and RIPK2, a key mediator of both innate immune signaling and adaptive immunity. The cleavage of RIPK1 or RIPK2 was not observed in cells treated with apoptotic stimuli, or cells stimulated with agonists to TNF-R1, nucleotide oligomerization domain receptor 1(NOD1), NOD2, Toll-like receptor 2 (TLR2) or TLR4. *P. gingivalis*-induced cleavage of RIPK1 and RIPK2 was inhibited in the presence of a lysine-specific gingipain (Kgp) inhibitor. RIPK1 and RIPK2 cleavage was not observed in HAEC treated with an isogenic mutant deficient in the lysine-specific gingipain, confirming a role for Kgp in the cleavage of RIPK1 and RIPK2. Similar proteolysis of poly (ADP-ribose) polymerase (PARP) was observed. We also demonstrated direct proteolysis of RIPK2 by *P. gingivalis* in a cell-free system which was abrogated in the presence of a Kgp-specific protease inhibitor. Our studies thus reveal an important role for pathogen-mediated modification of cellular kinases as a potential strategy for bacterial persistence within target host cells, which is associated with low-grade chronic inflammation, a hallmark of pathogen-mediated chronic inflammatory disorders.

## Author Summary

A number of successful pathogens have evolved mechanisms to evade host defenses, thus establishing persistent and chronic infections. Although membrane-bound innate immune receptors including Toll-like receptors play a role in inflammation in response to the common oral pathogen *Porphyromonas gingivalis*, it is not known how intracellular host defense mechanisms to this pathogen contribute to persistent infection and resulting chronic inflammation. In this study, we have defined a novel strategy by which *P. gingivalis* modulates the levels of key intracellular proteins involved in cell death and host defense responses. We demonstrate that the lysine-specific bacterial cysteine protease of *P. gingivalis* (Kgp) induces the proteolysis of receptor interacting protein kinase 1 (RIPK1), RIPK2 and poly (ADP-ribose) polymerase (PARP). Although endogenous host mechanisms may contribute to this process, activation of innate immune signaling cascades, caspases, or apoptosis alone were not sufficient to drive proteolysis. These findings support a role for pathogen-mediated modification of cellular kinases as a strategy for bacterial persistence within target host cells. Together with other recently described mechanisms for *P. gingivalis* host immune evasion, our work supports the emerging concept that pathogen-mediated chronic inflammatory disorders result from specific pathogen-mediated evasion strategies resulting in low-grade chronic inflammation.

## Introduction

RIPK1 and RIPK2 belong to a novel class of kinases that function in cell survival and cell death mechanisms [1], [2], [3]. The serine/threonine kinases share a conserved kinase domain and have distinct protein-protein interaction motifs, including a death domain in RIPK1 or a caspase activation and recruitment domain (CARD) in RIPK2. RIPK1 and RIPK2 participate in distinct cellular responses. RIPK1 is primarily involved in mediating TNF-R1-induced cell activation, apoptosis and necroptosis [4]. In contrast, RIPK2 functions as a key signaling protein in host defense responses induced by activation of the cytosolic pattern recognition receptors (PRR) NOD1 and NOD2 which sense the evolutionarily conserved bacterial peptidoglycan motifs, including *meso*-diaminopimelic acid (iE-DAP) and muramyl dipeptide (MDP) [5], [6], [7], [8], [9], [10], [11], [12]. RIPK2 has also been reported to mediate other cellular responses, including TLR signaling [13], [14], [15], [16], caspase 1 activation [17], apoptosis [1] and autophagy [18].

Increasing evidence demonstrates that both TNF-R1 and NOD-like receptor (NLR) pathways are regulated by and activate similar downstream signaling proteins [3], [16], [19], [20], [21]. However, the mechanisms that distinguish if RIPK1 and RIPK2 promote either cell survival or cell death signaling pathways are not fully understood. Induction of apoptosis through activation of death receptors (TNF-R1, Fas, DR3, DR4 or DR5) promotes the caspase-mediated cleavage of RIPK1 resulting in the generation of biologically active fragments with distinct functions that modulate NF-κB-mediated cell survival or death [22], [23]. A similar caspase-mediated cleavage of active fragments is observed with the cognate pronecrotic kinase RIPK3 and RIPK4 [24], [25]. The ability of particular protein motifs of RIPK1 to modify cell fate may be governed by post-translational modifications [19], [26] and the ability of death receptors to distinguish between various adaptor proteins [27].

Periodontitis is a destructive chronic inflammatory disease of the oral cavity that is mediated by a robust immune response to infection with pathogenic bacteria. Among the community of microorganisms associated with periodontitis, the Gram-negative anaerobe, *P. gingivalis* is a key etiologic agent of the disease. In addition to chronic inflammation at the initial site of infection, mounting evidence has accumulated supporting a role for *P. gingivalis*-mediated periodontal disease as a risk factor for systemic diseases including diabetes, pre-term birth, and cardiovascular disease [28], [29]. Dissemination of *P. gingivalis* from the oral cavity in humans is documented and *P. gingivalis* has been detected in human atheromas [30].

Animal studies have provided some of the strongest evidence for the role of *P. gingivalis* in the contribution of atherosclerosis. Our laboratory and others have demonstrated that oral challenge of *ApoE^−/−^* mice with *P. gingivalis* induces alveolar bone loss and increases the mean area and extent of atherosclerotic lesion development in the aortic arch in mice maintained on a normal chow diet [31], [32]. Aortic tissue analysis from *P. gingivalis*-challenged *ApoE^−/−^* mice demonstrate increased inflammatory markers and activation of the endothelium, including increased numbers of monocytes and elevated levels of cell adhesion molecules (CAM), TLR and expression of cytokines [31], [32], [33], [34]. Furthermore, high titers of systemic *P. gingivalis*-specific antibodies are generated and detection of *P. gingivalis*-specific 16S DNA by PCR in blood and aortic tissues has been observed early after oral challenge of *ApoE^−/−^* mice [31], [32], [33].

*In vitro* studies have established that *P. gingivalis* invades and survives within endothelial cells which is associated with the activation of the endothelium. *P. gingivalis* activation of endothelial cells is associated with up regulation of proinflammatory genes [33], elevation in cell surface expression of CAM [33], [35], [36], [37], TLR [31], [38], and the secretion of chemokines [33], [37], [39]. Following major fimbriae-mediated attachment, minor fimbriae play a role in more intimate attachment facilitating uptake [37]. *P. gingivalis* uptake into endothelial cells occurs quickly (30 min) [36], [37], [40], and upon entry *P. gingivalis* traffics to and resides in a late autophagosome [40]. The ability of *P. gingivalis* to both persist within endothelial cells and to activate inflammatory cascades has been postulated to play a role in low grade chronic inflammation associated with *P. gingivalis* infection [37]. *P. gingivalis* has a number of elaborate mechanisms which are utilized to evade detection and eradication by the immune system, including modification of lipid A, the biological core of bacterial LPS, and through activities associated with bacterial cysteine proteases (referred to as gingipains) [35], [37], [39]. The gingipains are transcribed from three genes, *rgpA*, *rgpB*, and *kgp*, producing arginine (R)-specific (RgpA and RgpB) or lysine-specific (Kgp) gingipains, that cleave after an arginine or a lysine residue, respectively [41], [42], [43]. The gingipains are non-covalently attached to the bacterial surface and can be secreted in vesicle-bound molecules or as monomers [41], [44]. Importantly, gingipains cleave a number of host cell proteins to facilitate *P. gingivalis* invasion [39], survival and colonization [45], [46].

In this study, we demonstrate that *P. gingivalis* infection of HAEC results in the rapid cleavage of RIPK1 and RIPK2, key mediators of cell survival, death and host defense. Our findings reveal that proteolysis of intracellular signaling kinases by bacterial lysine-specific proteases alters host defense responses and provides a potential mechanism for bacterial persistence within target host cells. Together with other recently described mechanisms for *P. gingivalis* host immune evasion, our work supports the emerging concept that pathogen-mediated chronic inflammatory disorders result from specific pathogen-mediated evasion strategies associated with low grade chronic inflammation.

## Results

### Induction of RIPK1 and RIPK2 proteolysis in HAEC treated with *P. gingivalis*

**Figure 1:**
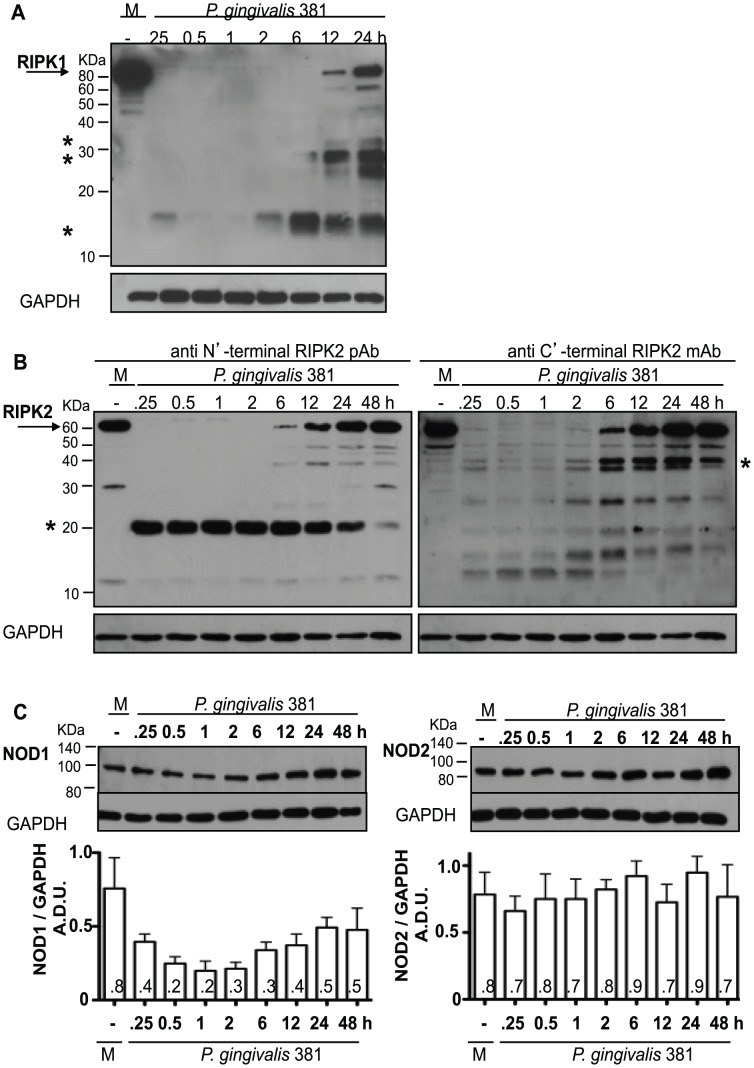
*P. gingivalis* 381-induced proteolysis of RIPK1 and RIPK2 in HAEC. HAEC were treated with medium (**M**) or with *P. gingivalis* strain 381 (MO1 100) for 0.25, 0.5, 1, 2, 6, 12, 24 or 48 h. Whole cell lysates were analyzed for the detection of **A**) RIPK1, **B**) RIPK2 with an anti N′-terminal RIPK2 antibody (left panel) or an anti C′-terminal RIPK2 antibody (right panel), or **C**) NOD1 (left panel) and NOD2 (right panel). Full-length RIPK1 (74-kDa) and RIPK2 (61-kDa) are indicated with arrows. Prominent *P. gingivalis*-induced LMW bands are indicated with asterisks. Molecular weight (MW) ladder is indicated on the left in kDa. GAPDH was detected as a loading control. (−) protein levels in medium-treated cells were similar at all time points. Densitometric analysis is presented below respective blots as the mean (+/− SEM) ratio of NOD1 (or NOD2) to GAPDH protein levels (arbitrary densitometric units (A.D.U.) from at least 3 independent membranes. Means are displayed within the bar charts.

**Figure 2:**
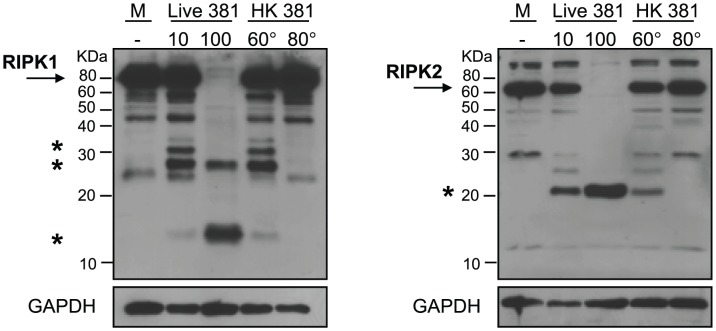
*P. gingivalis*-induced proteolysis of RIPK proteins is dose-dependent and heat labile. HAEC were treated with medium (**M**), live *P. gingivalis* strain 381 (MOI 10) (**10**), live *P. gingivalis* (MOI 100) (**100**), heat-killed (HK) (60°C, 60 min) *P. gingivalis* 381 (MOI 100 equivalency) (**60°**), or with HK (80°C, 20 min) *P. gingivalis* 381 (MOI 100 equivalency) (**80°**) for 2 h. Whole cell lysates were analyzed for the detection of RIPK1 (left panel) or RIPK2 (right panel). Full-length RIPK1 and RIPK2 are indicated with arrows. Prominent *P. gingivalis*-induced LMW bands are indicated with asterisk(s). MW ladder is indicated on the left in kDa. GAPDH was detected as a loading control.

We previously demonstrated that *P. gingivalis* induces the activation (defined as an up regulation of cell surface CAM, TLR, activation of NF-κB and secretion of chemokines, including IL-8 and MCP-1) of primary HAEC and human umbilical vein endothelial cells (HUVEC) in tissue culture [33], [35], [37], [39], [47] and acceleration of aortic lesion development in mouse models of *P. gingivalis* infection via TLR mediated signaling [31], [34], [38]. To begin to address the role of *P. gingivalis*-mediated intracellular signaling responses in HAEC, we first examined the expression level of the intracellular kinases RIPK1 and RIPK2. HAEC were treated with live organism at a multiplicity of infection (MOI) of 100 and protein levels were monitored over time. Target sites of antibodies to RIPK1 and RIPK2 used in this study are described in **Figure S1**. Treatment of HAEC with *P. gingivalis* strain 381 induced an immediate (15 min) and significant reduction of full-length RIPK1 and RIPK2 protein levels as determined by Western blot analysis of whole cell lysates (**Figure 1A and B**). The reduction of full-length RIPK1 and RIPK2 in HAEC was transient since full-length protein levels were observed at later time points. We also observed prominent low molecular weight (LMW) immunoreactive bands in samples treated with *P. gingivalis* with an antibody to RIPK1 (14-, 28- and 32-kDa) (**Figure 1A** and **Figure 2**), and with antibodies directed to the N′-terminal and C′-terminal region of RIPK2 (20- and 42-kDa), respectively (**Figure 1B**). Noteworthy, the sum of the immunoreactive bands observed for RIPK1 and RIPK2 was approximate to the molecular weight of RIPK1 (74-kDa) and RIPK2 (61-kDa), respectively. The detection of multiple bands with the RIPK2 C′-terminal antibody (**Figure 1B**-right panel) may represent fragments of additional proteolytic processing of RIPK2 following an initial cleavage event. Similar findings were observed in HUVEC (data not shown). In contrast to RIPK1 and RIPK2, we did not observe LMW immunoreactive bands with antibodies to NOD1 or NOD2, the cytosolic PRR of RIPK2, in HAEC treated with *P. gingivalis*. However, NOD1 levels were decreased in response to *P. gingivalis* with an initial decrease in protein levels and a partial recovery later in the infection. NOD2 levels were relatively stable in response to *P. gingivalis* as to untreated HAEC. (**Figure 1C**).

The *P. gingivalis*-induced proteolysis of RIPK1 and RIPK2 was dose-dependent (**Figure 2**). To determine if live *P. gingivalis* was required for the reduction of RIPK1 and RIPK2, HAEC were treated with heat-killed (HK) preparations of *P. gingivalis* (60°C or 80°C). Partial proteolysis of RIPK1 and RIPK2 was observed in HAEC treated with *P. gingivalis* cultures performed at 60°C, but not at 80°C suggesting that the ability of *P. gingivalis* to degrade RIPK1 and RIPK2 was dependent on a heat-labile bacterial protein (**Figure 2**).

The *P. gingivalis* major fimbriae mediate attachment to cells by interacting with host cell surface integrins and membrane-bound PRR [48]. Interfering with this interaction inhibits the ability of *P. gingivalis* to invade host cells within tissue culture systems [35], [47]. To determine if the major fimbriae was required for the modification of RIPK1 and RIPK2 in endothelial cells, protein levels in HUVEC were compared in cells treated with either the *P. gingivalis* major fimbriae-deficient strain DPG3 to wild type strain 381. As expected *P. gingivalis* strain 381 induced a dose-dependent reduction of RIPK1 and RIPK2 levels and induction of LMW immunoreactive bands (**Figure S2**). Importantly, full-length RIPK1 and RIPK2 levels were not significantly reduced in endothelial cells treated with *P. gingivalis* strain DPG3. These observations suggest that the functional activity of *P. gingivalis* fimbriae contribute to the observed proteolysis of RIPK1 and RIPK2.

To determine if the LMW immunoreactive bands observed following incubation with *P. gingivalis* were products of new protein synthesis or a putative cleaved fragments of RIPK1 or RIPK2, HUVEC were pretreated with the protein synthesis inhibitor cycloheximide (CHX) prior to stimulation with *P. gingivalis*. Pretreatment with CHX did not alter the ability of *P. gingivalis* to induce the proteolysis of RIPK1 or RIPK2 (**Figure S3**). These findings indicate that the LMW immunoreactive bands observed in *P. gingivalis* treated cells, were not products of new protein synthesis and suggest that they are cleavage fragments of RIPK1 or RIPK2, respectively. Collectively, these results demonstrate that *P. gingivalis* induced the immediate, transient, dose-dependent proteolysis of RIPK1 and RIPK2 through a fimbriae-facilitated and heat-labile process.

### RIPK2 levels are stable in HAEC following stimulation with TLR or NLR agonists or following stimulation with TNFα

**Figure 3:**
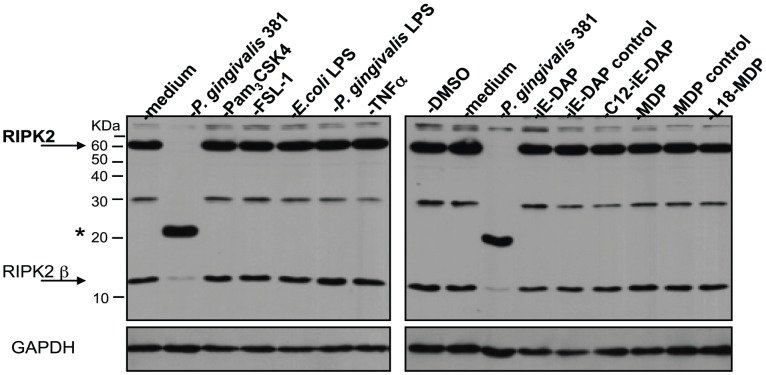
RIPK2 levels are stable in HAEC stimulated with TLR or NLR agonists. HAEC were treated with medium, *P. gingivalis* 381 (MOI 100), 10 µg/ml Pam_3_CSK4, 10 µg/ml FSL-1, 10 µg/ml *P. gingivalis* 381 LPS, 1.0 µg/ml *E. coli* 0111:B4 LPS, 100 ng/ml recombinant human TNF, 100 µg/ml iE-DAP, 100 µg/ml iE-DAP control, 1000 ng/ml C12-iE-DAP, 0.01% DMSO (C12-iE-DAP vehicle control), 100 µg/ml MDP, 100 µg/ml MDP control, or 1000 ng/ml L18-MDP for 2 h. Whole cell lysates were analyzed for the detection of RIPK2 and GAPDH. Full-length RIPK2 and RIPK2β are indicated with arrows. A prominent *P. gingivalis*-induced LMW band is indicated with an asterisk. MW ladder is indicated on the left in kDa.

To determine if *P. gingivalis*-induced proteolysis of RIPK2 was a result of cell activation via TLR or NLR-mediated signaling, HAEC were treated with a panel of synthetic or purified agonists to PRR that have been demonstrated to be activated by *P. gingivalis* or are direct activators of the NOD/RIPK2 signaling pathway. HAEC were untreated or treated with live *P. gingivalis* or with agonists to PRR, including Pam_3_CSK4, FSL-1, *P. gingivalis* LPS, *E. coli* LPS, iE-DAP, MDP or TNFα as a non-PRR activator of NF-κB or their vehicle controls for 2, 6, 12 and 24 h. To facilitate the passage of iE-DAP or MDP through the cell membrane, acylated derivatives of iE-DAP (C12-iE-DAP) and MDP (L18-MDP) were also used. Stimulation of HAEC with the panel of agonists did not result in the proteolysis of full-length RIPK2 or its mRNA splice variant, RIPK2β [49] (**Figure 3**). Similar results were obtained using a 100-fold range for all agonists examined over the full time course of the experiment (data not shown). Treatment of HAEC with the agonists resulted in the induction of IL-8 levels in a manner dependent on the agonist and concentration used (**Figure S4**). Noteworthy, NOD1 agonists (iE-DAP and C12-iE-DAP) induced significantly higher IL-8 levels in comparison to NOD2 agonists (MDP, L18-MDP), TLR2, and TLR4 agonists. Collectively, these findings suggest that the modification of RIPK2 observed in HAEC treated with *P. gingivalis* was not simply due to the activation of a single TLR, NOD or TNF-R1 canonical signaling pathway but a response driven by *P. gingivalis*.

### The proteolysis of RIPK1 and RIPK2 does not a result from the induction of apoptosis

**Figure 4:**
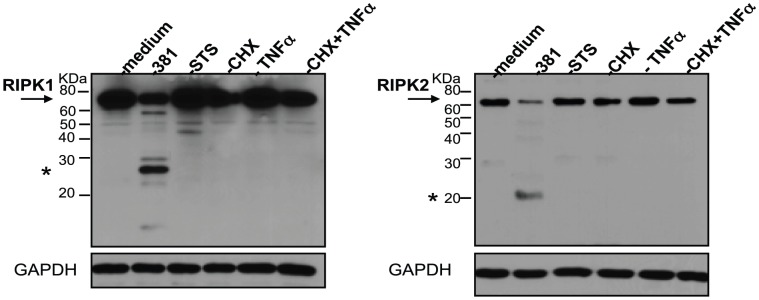
Classical apoptotic stimuli do not induce the proteolysis of RIPK1 or RIPK2 in HUVEC. HUVEC were treated with medium, *P. gingivalis* 381 (MOI 100), 2 µM staurosporine (**STS**) 25 µg/ml cycloheximide (**CHX**), 10 ng/ml TNFα, or co-treated with 25 µg/ml CHX and 10 ng/ml TNFα for 6 h. Whole cell lysates were analyzed for the detection of RIPK1 (left panel) or RIPK2 (right panel). Full-length RIPK1 and RIPK2 are indicated with arrows. Prominent *P. gingivalis*-induced LMW bands are indicated with asterisks. MW ladder is indicated on the left in kDa. GAPDH was detected as a loading control.

To determine if *P. gingivalis*-induced proteolysis of RIPK1 and RIPK2 was a product of apoptosis, we treated cells with apoptotic stimuli and monitored apoptosis by flow cytomery detection of annexin V/propidium iodide staining and by Western blot detection of cleaved caspase 3 and its substrate PARP. Treatment of HUVEC with staurosproine or co-treatment with CHX and TNFα (CHX+TNFα) induced significant apoptotic cell populations as compared to HUVEC treated with CHX alone, TNFα alone or medium (**Figure S5A**). We also observed dose-dependent apoptotic cell populations treated with *P. gingivalis* 381, suggesting that a proportion of the cells undergo apoptosis in response to infection. Induction of apoptosis of HUVEC by staurosporine or CHX+TNFα treatment was also confirmed through the cleavage of caspase 3 and PARP (**Figure S5B**). Importantly, we did not observe the induction of RIPK1 or RIPK2 cleavage in HUVEC in response to staurosporine or CHX+TNFα treatment (**Figure 4**). Furthermore, *P. gingivalis* itself did not induce the cleavage of caspase 3, but appeared to induce the complete proteolysis of PARP. These findings demonstrate that apoptotic stimuli alone do not induce the cleavage of RIPK1 or RIPK2 in HUVEC.

### Role of caspases in *P. gingivalis*-induced proteolysis of RIPK1 and RIPK2

**Figure 5:**
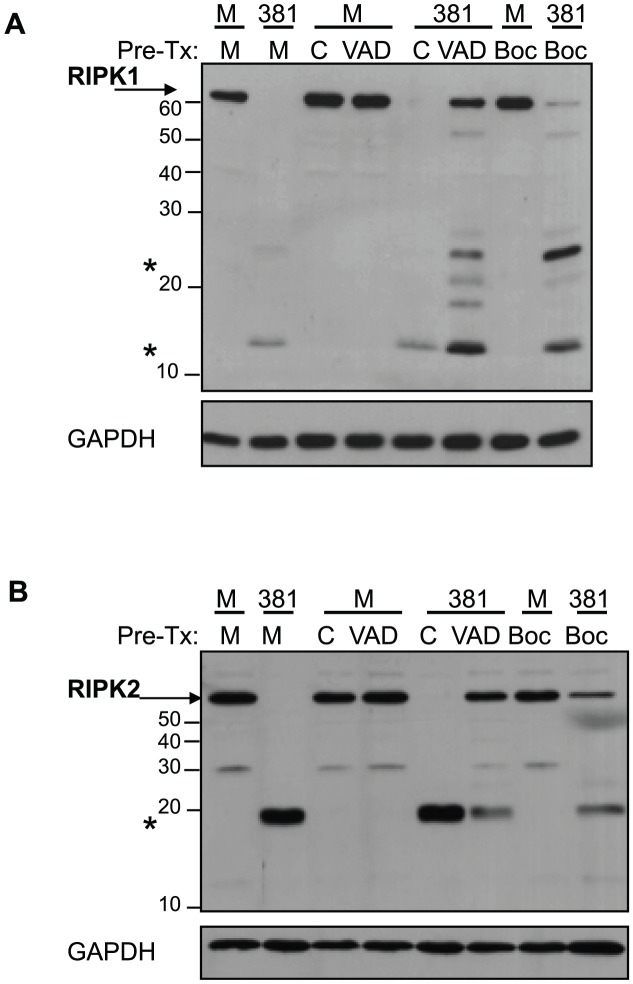
General caspase inhibitors z-VAD-FMK and Boc-D-FMK alter *P. gingivalis*-induced modification of RIPK1 and RIPK2 in HUVEC. HUVEC were pretreated (Pre-Tx) with medium (**M**), 0.25% DMSO vehicle control (**C**), 25 µM z-VAD-FMK (**VAD**), or 100 µM Boc-D-FMK (**Boc**) with for 1.5 h. HUVEC were then treated with medium (**M**) or *P. gingivalis* strain 381 (MOI 100, **381**) for 2 h. Whole cell lysates were analyzed for (**A**) RIPK1 or (**B**) RIPK2 and GAPDH. Full-length RIPK1 and RIPK2 are indicated with arrows. Prominent *P. gingivalis*-induced LMW bands are indicated with asterisks. MW ladder is indicated on the left in kDa.

Previous reports have identified a caspase-mediated cleavage of RIPK1, RIPK3 and RIPK4 during death receptor-mediated apoptosis [22], [23], [24], [25]. Despite these reports, prior to our study there have been no reports demonstrating the cleavage of RIPK2 under any condition. To determine if caspase inhibitors prevented the proteolysis of RIPK1 and RIPK2 in cells treated with *P. gingivalis*, HUVEC were pretreated with the general caspase inhibitors zVAD-FMK, Boc-D-FMK or vehicle control prior to addition of live organism to the cell culture. We confirmed that the general caspase inhibitors abrogated caspase activity as monitored by inhibition of staurosporine-induced PARP cleavage (Figure S6-*lower panel*). Treatment of cells with general caspase inhibitors interfered with *P. gingivalis*-mediated proteolysis of RIPK1 (**Figure 5A** **, Figure S6**-top panel) and RIPK2 (**Figure 5B** **and Figure S6**-mid panel). While these findings suggest that caspases are involved in the proteolysis of RIPK2, FMK-linked general and enzyme-specific caspase inhibitors have been demonstrated to inhibit the proteolytic activity of the *P. gingivalis* cysteine protease Kgp, but not the arginine-specific gingipains [50]. Importantly, both caspases and gingipains are classified under the same clan of cysteine proteases (C25), as they share similar active sites, structural motifs and are subject to similar modes of chemical inhibition [51]. Thus, the ability of caspase inhibitors to interfere with *P. gingivalis*-induced RIPK2 proteolysis by caspases inhibitors may reflect an inhibition of host caspase activity, *P. gingivalis* Kgp activity, or both.

**Figure 6:**
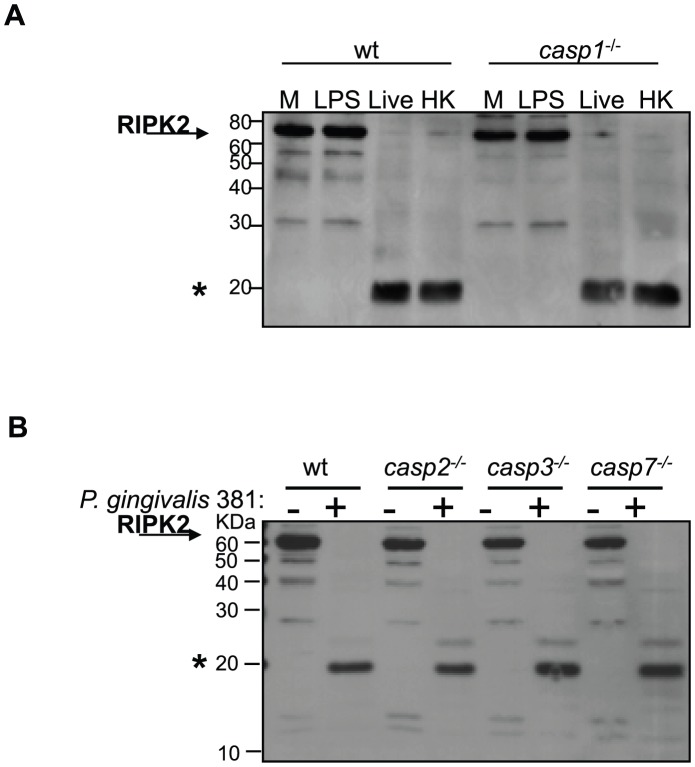
*P. gingivalis* modifies RIPK2 in wild type and caspase-deficient murine bone marrow-derived macrophages. **A**) C57BL/6 (**wt**) or *casp1*-deficient (***casp1^−/−^***) BMDM were untreated (**M**) or treated with 100 ng/ml *E. coli* LPS (**LPS**), live *P. gingivalis* 381 (MOI 100, **Live**) or heat-killed (60°C, 60 min) *P. gingivalis* 381 (MOI 100 equivalency, **HK**) for 2 h. **B**) C57BL/6 (**wt**), *casp2*-deficient (***casp2^−/−^***), *casp3*-deficient (***casp3^−/−^***), or *casp7*-deficient (***casp7^−/−^***) BMDM were untreated (**−**) or treated with *P. gingivalis* 381 (MOI 100) (**+**) for 2 h. Whole cell lysates were analyzed for RIPK2. Full-length RIPK2 is indicated with an arrow. A prominent *P. gingivalis*-induced LMW band is indicated with an asterisk. MW ladder is indicated on the left in kDa.

To examine the role of cellular caspase activity in *P. gingivalis*-induced proteolysis of RIPK2 directly, murine bone marrow derived macrophages (BMDM) from *casp1*-, *casp2*-, *casp3*-, or *casp7*-deficient mice were co-cultured with *P. gingivalis*. *P. gingivalis* induced a similar proteolysis of RIPK2 in cells deficient in *casp1* (**Figure 6A**), *casp2*, *casp3*, or *casp7* (**Figure 6B**) as that observed in wild type cells. These findings suggest that caspase 1, caspase 2, caspase 3 and caspase 7 singly do not play a role in *P. gingivalis*-induced RIPK2 proteolysis. However, they do not exclude the possibility that non-redundant host caspase activity could contribute to RIPK2 cleavage induced by *P. gingivalis* stimulation.

### *P. gingivalis* 381-induced proteolysis of RIPK1 and RIPK2 is abrogated with peptide inhibition of Kgp

**Figure 7:**
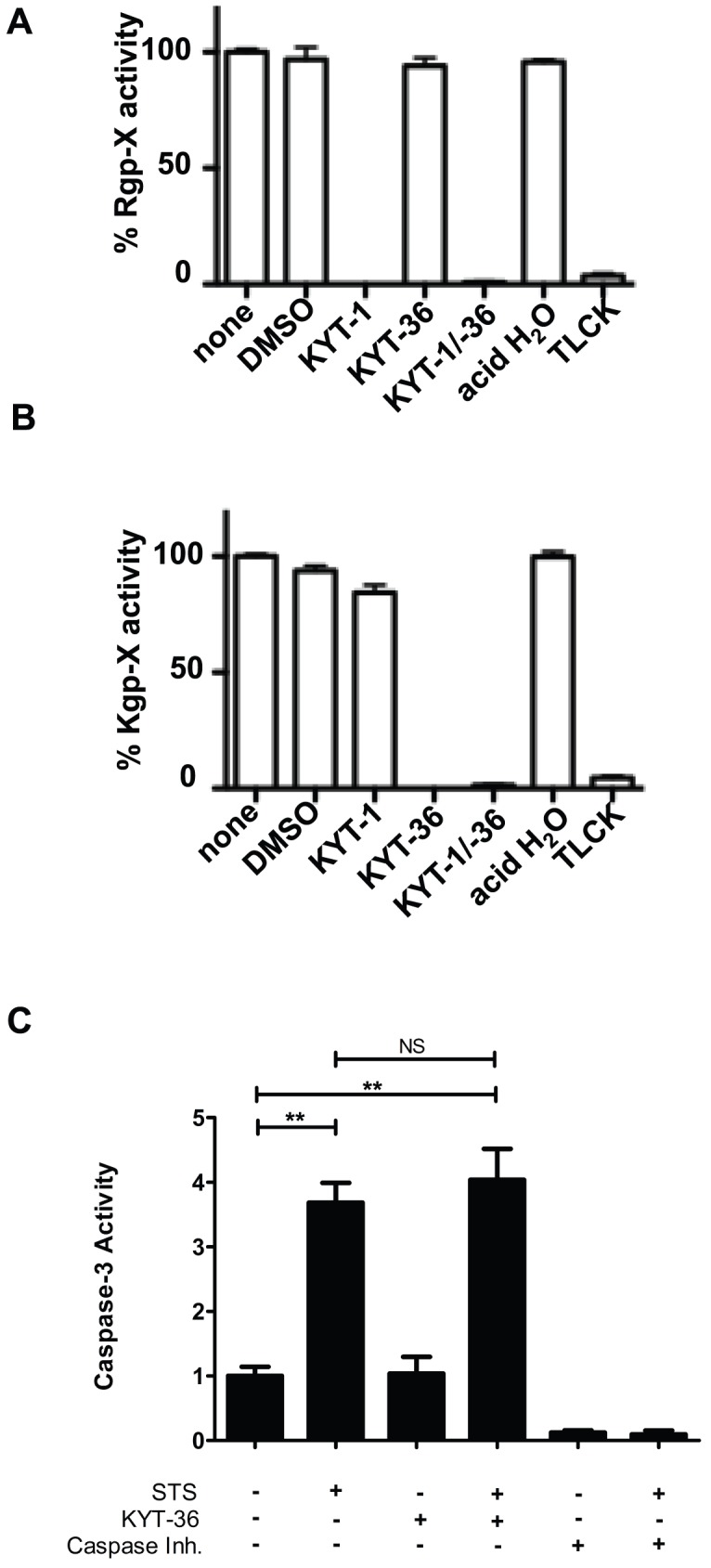
KYT inhibitors specifically inhibit *P. gingivalis* gingipain activity and do not alter host 3 caspase activity. Effect of gingipain inhibitors on *P. gingivalis* **A**) Rgp or **B**) Kgp protease activity. *P. gingivalis* was untreated (none) or pretreated with 10 µM Rgp-specific inhibitor KYT-1, 10 µM Kgp-specific inhibitor KYT-36, 10 µM KYT-1 and 10 µM KYT-36, 1 mM TLCK, or vehicle controls (DMSO or acid water) for 10 min and monitored for arginine-X-specific or lysine-X-specific protease activity. Effect of inhibitors on *P. gingivalis* is presented as percent Rgp-X activity or Kgp-X activity relative to untreated *P. gingivalis*. **C**) HUVEC were untreated or treated with 2 µM staurosporine (STS) for 5 h. Whole cell lysates were analyzed for caspase-3 activity in the presence of KYT-36 gingipain inhibitor (3 µM). Activity is represented as fold change relative to untreated. A reversible caspase inhibitor was included to demonstrate observed fluorescence is specific to caspase-3 like proteases. Statistical analysis was performed using unpaired T-test (α = 0.05), **p<0.001, NS = no significance.

**Figure 8:**
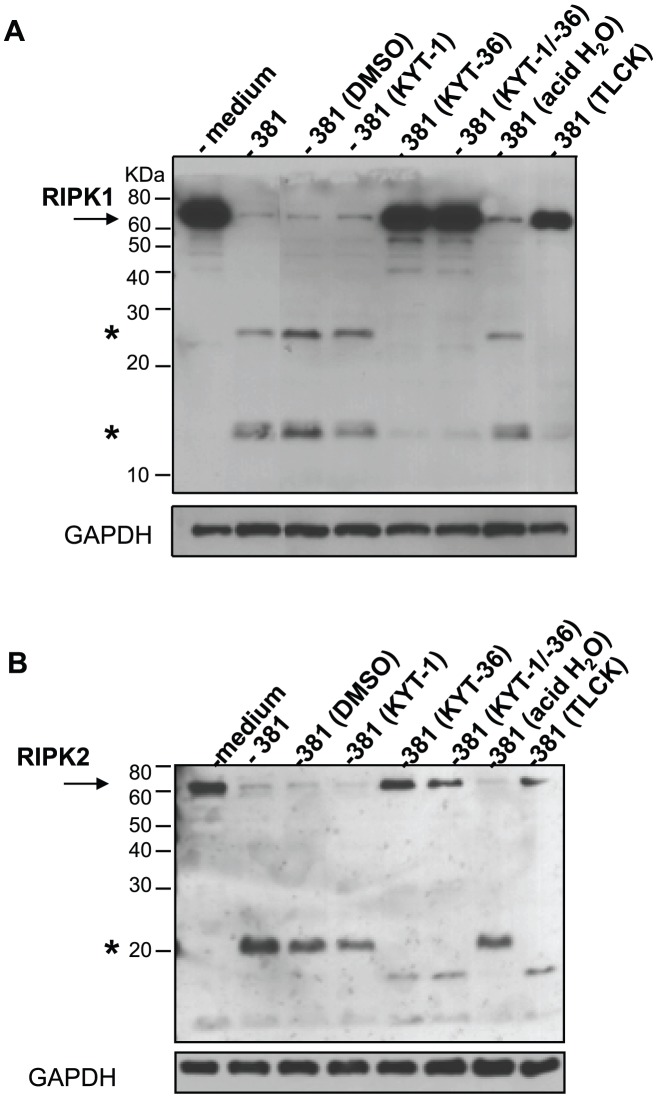
Inhibition of Kgp activity alters *P. gingivalis*-mediated RIPK1 and RIPK2 cleavage in HAEC. *P. gingivalis* strain 381 was pretreated with 10 µM KYT-1, 10 µM KYT-36, 10 µM KYT-1 and 10 µM KYT-36, 1 mM TLCK, or vehicle controls (DMSO or acid water) for 45 min. HAEC were then immediately co-cultured with medium or with pretreated preparations of *P. gingivalis* 381 (MOI 100) for 2 h. Whole cell lysates were analyzed for **A**) RIPK1 or **B**) RIPK2. Full-length RIPK1 and RIPK2 are indicated with arrows. Prominent *P. gingivalis*-induced LMW bands are indicated with asterisk(s). MW ladder is indicated on the left in kDa. GAPDH was detected as a loading control.

A number of host cell surface proteins and proteins in the extracellular milieu are cleaved by the cysteine proteases of *P. gingivalis* [44], [52], [53]. To evaluate if *P. gingivalis* gingipains played a role in the proteolysis of RIPK1 and RIPK2, we first evaluated previously established gingipain-specific inhibitors KYT-1 (arginine-specific gingipain inhibitor) and KYT-36 (lysine-specific gingipain inhibitor) [54], [55] as well as TLCK, which inhibits both arginine-specific and lysine-specific protease activity [56] in our studies. We confirmed the ability of the protease inhibitors to reduce the arginine-specific activity by KYT-1, the lysine-specific activity by KYT-36 and both arginine- and lysine-specific protease activity by TLCK in a substrate-based assay (**Figure 7A and B**). Treatment of *P. gingivalis* 381 with KYT-1 or vehicle control (DMSO) did not alter the ability of *P. gingivalis* 381 to induce proteolysis of RIPK1 (**Figure 8A**) or RIPK2 (**Figure 8B**) in HAEC. However, treatment of *P. gingivalis* with KYT-36 alone or in combination with KYT-1 blocked *P. gingivalis*-induced proteolysis of RIPK1 and RIPK2. TLCK also inhibited the *P. gingivalis*-induced proteolysis of RIPK1 and RIPK2. Pretreatment of *P. gingivalis* with KYT-1, KYT-36, TLCK and vehicle controls did not alter bacterial viability (data not shown), nor did KYT-36 alter host caspase 3 activity (**Figure 7C**). These findings indicate that Kgp activity was responsible for *P. gingivalis*-mediated proteolysis of RIPK1 and RIPK2.

### RIPK1 and RIPK2 levels in HAEC are unaltered with the *P. gingivalis kgp*-deficient mutant strain YPP2

**Figure 9:**
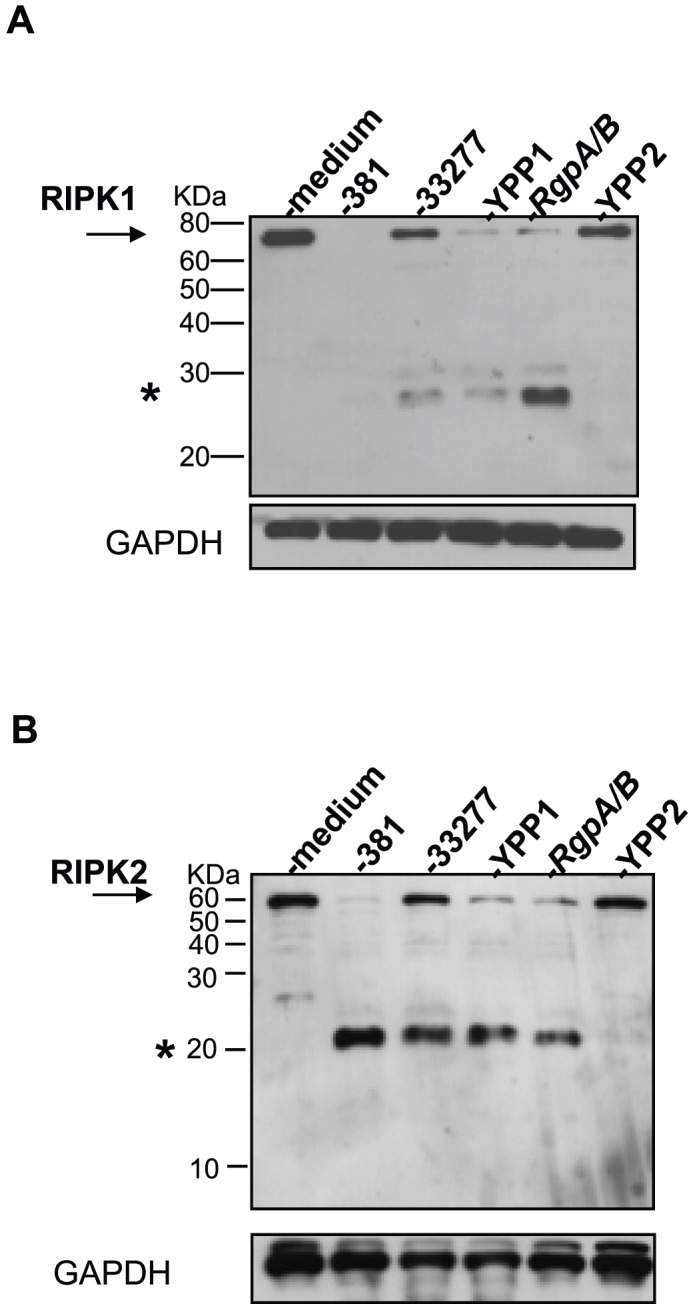
*P. gingivalis* Kgp mutant is deficient in the induction of RIPK1 and RIPK2 proteolysis in HAEC. HAEC were untreated or treated with *P. gingivalis* strain 381, strain ATCC 33277, or isogenic mutants of 33277: YPP1 (*rgpA^−^*), *RgpA/B* (*rgpA^−^*, *rgpB^−^*), or with YPP2 (*kgp^−^*) (MOI 100) for 2 h. Whole cell lysates were analyzed for **A**) RIPK1 or **B**) RIPK2. Full-length RIPK1 and RIPK2 are indicated with arrows. Prominent *P. gingivalis*-induced LMW bands are indicated with asterisks. MW ladder is indicated on the left in kDa. GAPDH was detected as a loading control.

To further confirm the role of *P. gingivalis* gingipains in the proteolysis of RIPK1 and RIPK2 in the absence of chemical inhibitors, we examined previously characterized bacterial mutant strains deficient in *kgp* or *rgp* [57] for their ability to induce the proteolysis of RIPK1 and RIPK2. The genetic background of the mutant strains used was in *P. gingivalis* strain 33277, a similarly classified organism in terms of clonality (clonal type 1) [58] serogroup (A) [59] and invasiveness [60] as *P. gingivalis* strain 381. We observed that *P. gingivalis* wild type strain 33277 reduced full-length RIPK1 (**Figure 9A**) and RIPK2 (**Figure 9B**) levels and induced the detection of LMW immunoreactive bands. However, the degree of RIPK1 and RIPK2 proteolysis induced by 33277 was less than that observed with strain 381. Both *P. gingivalis* strains YPP1 and RgpA/B induced a significant proteolysis of full-length RIPK1 and RIPK2. In contrast, RIPK1 and RIPK2 protein levels in HAEC treated with *P. gingivalis* YPP2 were similar to untreated cells. These findings confirm the role of Kgp in *P. gingivalis*-induced proteolysis of RIPK1 and RIPK2.

### *P. gingivalis* Kgp activity induces the proteolysis of PARP

Initially monitoring the cleavage of the nuclear DNA repair enzyme PARP [61], [62], [63] as a marker of caspase-mediated apoptosis, we serendipitously observed that *P. gingivalis* induced the proteolysis of PARP in the absence and presence of broad-based caspase inhibition (**Figure S6**). The kinetics of PARP levels in response to *P. gingivalis* followed an immediate proteolysis (15 min) that was sustained until 24 h of co-culture when PARP levels began to return to baseline (**Figure S7A**). We noted the proteolysis of PARP was dose-dependent and that the absence of PARP detection was noted in both soluble and insoluble cellular fractions (data not shown). We thus further evaluated the role of gingipain activity on the proteolysis of PARP through chemical inhibition or through the use of isogenic mutants lacking gingipains as described above for RIPK1 and RIPK2. We observed a similar pattern of *P. gingivalis*-induced proteolysis of PARP as to that observed with RIPK1 and RIPK2 when using specific gingipain inhibitors or bacterial mutants (**Figure S7B and C**). These findings suggest that not only are RIPK1 and RIPK2 proteins subject to Kgp-induced proteolysis, but other key regulatory proteins involved in regulating apoptosis (such as PARP) may be subject to similar proteolysis in response to *P. gingivalis*.

### *P. gingivalis* induces the cleavage of recombinant RIPK2 kinase domain independent of host cell proteins

**Figure 10:**
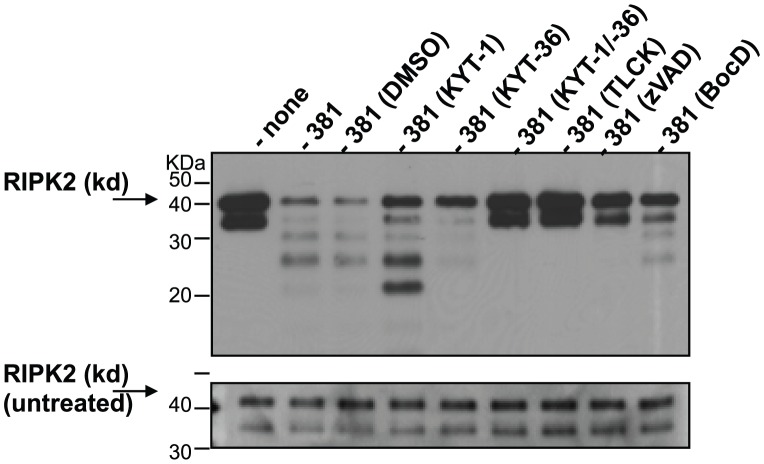
Cleavage of recombinant RIPK2 kinase by *P. gingivalis* in the absence of host cell proteins. *P. gingivalis* strain 381 was pretreated with 10 µM KYT-1, 10 µM KYT-36, 10 µM KYT-1 and 10 µM KYT-36, 1 mM TLCK, 100 µM zVAD-fmk, 100 µM BocD-fmk with or vehicle controls (HEPES (none), DMSO or acid water) for 45 min, then immediately co-cultured with 0.1 µg recombinant RIPK2 kinase for 1 h at 37°C. Reactions were stopped by the addition of SDS-PAGE loading dye and analyzed by Western blot analysis with an antibody to the N′-terminal kinase domain of RIPK2. Top panel: reaction with recombinant protein and *P. gingivalis*; bottom panel: 10% of reaction prior to incubation with *P. gingivalis* (untreated recombinant protein, i.e., gel loading control).

To determine if *P. gingivalis* was capable of cleaving RIPK2 in the absence of host cell proteins, recombinant RIPK2 kinase domain (rRIPK2 kd) was incubated with *P. gingivalis* and analyzed by Western blot for the detection of cleavage products with an antibody targeting the N′-terminal kinase domain. As shown in **Figure 10**, rRIPK2 kd migrates at ∼40-kDa and was cleaved in the presence of *P. gingivalis* 381. Furthermore, KYT-1 partially inhibited *P. gingivalis*-induced cleavage of RIPK2 kd. Surprisingly, the lowest molecular weight band was of a similar sized fragment ∼20-kDa as observed in endothelial cells treated with *P. gingivalis* (**Figure 1B**-left panel). Proteolysis of rRIPK2 kd was further inhibited with KYT-36, and completely inhibited in the presence of both KYT-1 and KYT-36, or with TLCK. We also observed that pretreatment of *P. gingivalis* with caspase inhibitors prevented proteolysis, with zVAD-fmk activity having a greater effect than BocD-fmk activity. The ability of these peptide inhibitors to prevent *P. gingivalis*-induced proteolysis of RIPK2 in a cell-free system is similar to our findings observed in endothelial cell culture. These findings support the notion that *P. gingivalis* may bypass cellular activation mechanisms and directly induce the proteolysis of RIPK2 and other intracellular proteins via Kgp-specific cleavage.

## Discussion

In this study, we demonstrate that the *P. gingivalis* lysine-specific cysteine protease, Kgp, induced the novel proteolysis of RIPK1, RIPK2 and PARP in human endothelial cells. The proteolysis of RIPK1 and RIPK2 reduced full-length protein levels and generated low molecular weight fragments. *P. gingivalis*-induced proteolysis was dose-dependent and inhibited through heat inactivation. In contrast, the induction of apoptosis of human endothelial cells by classical apoptotic stimuli did not induce the cleavage of RIPK1 or RIPK2. Furthermore, activation of canonical signaling pathways mediated by RIPK2 did not induce the proteolysis of RIPK2 with defined agonists to TLR2, TLR4, NOD1 and NOD2. Through the use of chemical inhibition and isogenic bacterial strains, we demonstrate the proteolysis of RIPK1, RIPK2 and PARP in endothelial cells was mediated specifically by *P. gingivalis* Kgp, and not by Rgp activity. In a cell-free assay we further demonstrated that *P. gingivalis* induced the direct proteolysis of recombinant RIPK2, which was inhibited in the presence of specific protease inhibitors. Our findings indicate that the proteolysis of RIPK1, RIPK2 and PARP induced by *P. gingivalis* can occur directly and does not require activation of innate immune signaling pathways or caspase-dependent apoptotic pathways.

The endothelial cell response to *P. gingivalis* infection has been described as that of an inflammatory nature with minimal apoptosis [33], [35], [36], [37], [39], [47]. In the current study we demonstrate that *P. gingivalis*-induced proteolysis of key proteins that mediate TNF-R superfamily-induced apoptosis, including RIPK1, RIPK2 and PARP [1], [64], [65], [66]. Previous reports demonstrated caspase-mediated cleavage of RIPK1, RIPK3 and RIPK4 in immortalized cell lines in response to death receptor activation [22], [23], [24], [25]. To our knowledge there have not been any reports demonstrating the cleavage of RIPK2 by induction of apoptosis or by a microbial pathogen. In contrast to the cleavage of RIPK family members in cell lines, we did not observe the cleavage of RIPK1 or RIPK2 in primary endothelial cells treated with similar apoptotic conditions. Furthermore, the size of the cleavage fragments of RIPK1 generated between Meylan and coworkers and those by *P. gingivalis* differed, which may be reflective of the cell types and mechanisms involved.

The degree of apoptosis (monitored by annexin V staining and caspase 3 cleavage) we observed by *P. gingivalis*-treated endothelial cells was minimal relative to the degree of RIPK1 and RIPK2 proteolysis at a given time point. The return of PARP levels, in particular, at later time points (24 h) during the infection supports the notion that *P. gingivalis* did not induce a caspase-mediated cell death program in endothelial cells, as *de novo* synthesis of PARP would not be expected in apoptotic cells. Although host factors may contribute to our observations, our findings with caspase inhibitors, caspase-deficient BMDM and cell-free assays suggest that the proteolysis of RIPK1, RIPK2 and PARP was not a caspase-mediated event, but was driven by the proteolytic activity of *P. gingivalis* Kgp. Virulence factors by other pathogens, including the catalytic activity of *Yersinia enterocolitica* outer protein P (YopP) and murine cytomegalovirus M45-encolded viral inhibitor of RIP activation (vIRA) have been shown to induce the caspase-mediated cleavage of RIPK1 or disruption of the pronecrotic RIPK1-RIPK3 complex, respectively, to promote cell death pathways [67], [68], [69]. Bacterial cell extracts or purified gingipains from *P. gingivalis* have been shown to induce endothelial cell apoptosis [50], [70]. In contrast, *P. gingivalis* Kgp-mediated cleavage of RIPK1 and RIPK2 in the context of viable whole bacteria may function to prevent activation of cell death pathways and serve to create an intracellular niche for bacterial persistence.

It is unclear if intracellular NLR activation contributes to host defense against live *P. gingivalis*. Bacterial components of *P. gingivalis* have been implicated to have a stimulatory effect of NOD1 and NOD2 in over-expression systems, and increased detection of NALP3 has been described in diseased human gingival tissue [71], [72], [73]. In contrast, the role of membrane-bound TLR in *P. gingivalis* infection and pathogenesis has been more thoroughly evaluated [33], [34], [35], [37], [39], [47], [74]. We previously demonstrated that *P. gingivalis* primes human endothelial cells for further TLR2 and TLR4 stimulation by inducing the up regulation of cell surface expression of TLR from intracellular stores [38], [48]. We also reported that *P. gingivalis* major and minor fimbriae induce IL-8 production in endothelial cells via TLR2 and TLR4/MD-2 signaling pathways [48]. TLR2 has also been demonstrated to mediate *P. gingivalis*-induced inflammatory bone loss *in vivo* [34], [75] and atherosclerotic lesion development in a *ApoE^−/−^* mouse model [34], [76].

An additional observation from these studies was the differential response by primary human endothelial cells to defined NOD and TLR agonists. We show that endothelial cells generated greater IL-8 secretion in response to NOD1 agonists as compared to that observed with TLR2, TLR4, and NOD2 agonists. Following stimulation with invasive bacteria, NOD1 and NOD2 translocate to the plasma membrane and recruit RIPK2 to mediate NF-κB and MAPK activation of host defenses [9], [77], [78]. Recent studies have also reported the importance of NOD1 in host defense by endothelial cells to intracellular bacteria, including *Chlamydophila pneumoniae*, *Listeria monocytogenes*, and *Orientia tsutsugamuschi* [6], [7], [79]. Although these reports did not evaluate the role of NOD1 on pathogen survival in endothelial cells, NOD1, NOD2 and RIPK2 have demonstrated to contribute to pathogen clearance [2], [3], [5], [9], [10], [12], [80]. Collectively, results presented here together with our previous studies indicate that *P. gingivalis* can both trigger TLR activation pathways while also interfering with the NOD1/RIPK2 cascade via rapid proteolysis of RIPK2.

The ability of *P. gingivalis* to persist and contribute to chronic disease may also result from disruptions in the intracellular signaling pathways that regulate autophagy. Autophagy involves the vesicular sequestration of organelles and the generation of autophagosomes to recycle amino acids and macromolecules. Autophagy can function as a cell survival mechanism, contribute to innate immune host defense by clearance of intracellular bacteria (xenophagy), and less often, contribute to cell death. Given the recent understanding of RIPK1, RIPK2 and PARP requirement in autophagy [4], [18], [81], our findings suggest that the ability of *P. gingivalis* to rapidly induce the proteolysis of RIPK1, RIPK2 and PARP in endothelial cells may provide a window of opportunity to escape the activation of xenophagy, invade and establish an intracellular niche. In support of this, it is well documented that *P. gingivalis* survives within primary endothelial cells [38], [39], [47]. Others and we have shown that *P. gingivalis* reside intracellularly in autophagosomes [40], [82], [83], [84]. Subversion of the autophagic pathway has been observed with other intracellular bacteria, including, *Brucella abortus* [85], *Legionella pneumophila* [86], and *Coxiella burnetii*. However, the role of *P. gingivalis* gingipain activity in modulating the autophagic machinery has not been elucidated. Noteworthy, in our studies we observed the return of full-length RIPK1, RIPK2 and PARP protein levels at later time points (24 h) following infection. Nonetheless, it is unclear if gingipain activity continues within the localized vacuole harboring *P. gingivalis* for the acquisition of nutrients or if the gingipains continue to target intracellular host proteins throughout the infection to regulate the balance of cell survival and death.

Historically, *P. gingivalis* gingipains were demonstrated to mediate proteolysis of extracellular and cell surface proteins and facilitate invasion of host tissues. The ability of *P. gingivalis* gingipains to induce the proteolysis of intracellular proteins in the absence of live bacteria is noteworthy. Gingipain-mediated cleavage of several intracellular structural (β-catenin, γ-catenin, p120, paxillin) and regulatory proteins (focal adhesion kinase, SRC, and p130CAS) have been reported with the use of live *P. gingivalis*, whole bacterial lysates of *P. gingivalis* and purified outer membrane vesicles (OMV) released by *P. gingivalis* (containing fimbriae, gingipains, LPS, capsule and muramic acid) [55], [87], [88], [89]. Importantly, the proteolysis of these intracellular proteins was dependent on *P. gingivalis* fimbriae. The subtle proteolysis of RIPK2 we observed with the *P. gingivalis fimA* mutant (DPG3) may be attributed to the poor invasion capacity of this organism. Upon re-evaluation of our HK preparations of *P. gingivalis* wild type strain 381, we observed that HK 60°C preparations maintained a significant level of Kgp and Rgp activity (data not shown). Similarly, the passage of fimbriae-containing OMV from *P. gingivalis* 381 with residual catalytic activity may have been sufficient to induce the partial proteolysis of RIPK1 and RIPK2. Thus, the ability of *P. gingivalis* OMV and gingipains to access intracellular host proteins is most likely enhanced with invasive, whole organism.

Recently, the subcellular distribution of NOD receptors has been further investigated. Using immunoflourescence and biochemical fractionation of tagged proteins, NOD2 has been shown to be localized to the plasma membrane, leading to recruitment of RIPK2 [90]. Both NOD1 and NOD2 are recruited to invasion foci of *Shigella flexneri* in HeLa cells [78], likely a mobilization strategy where the active complex is able to activate part of the NF-κB pathway via an “induced proximity” mechanism [91]. Considering RIPK2 recruitment occurred in a CARD-dependent manner, it is likely that NOD1 may similarly function in localization of RIPK2 to the plasma membrane, while RIPK1 recruitment occurs via TNF-R [92].

Several pathogens are internalized into host cells by clathrin-dependent endocytosis [93], which also contain NOD2 stimulatory activity. MDP entry appears to occur via a similar endocytic pathway, suggesting MDP and bacteria utilize the same route to trigger NOD2 activation [94]. Staphylococcal peptidoglycan is internalized via endocytosis and colocalizes with both NOD2 and TLR2, providing additional support for overlap in cellular distribution of PRR engagement of RIPK1 and RIPK2 in the vicinity of organism or OMV [95].

Previous work has established that for *P. gingivalis*, the majority of adherent bacteria actively invade endothelial cells, suggesting a highly efficient endocytic uptake of organisms [47]. We speculate that OMV can enter endothelial cells via similar mechanisms, such as receptor mediated uptake at particular lipid raft domains, as was shown in HeLa cells [55]; sites enriched in particular receptors and signaling molecules. As an early endosome forms and pinches off, initial localization of RIPK1 and RIPK2 on the plasma membrane now places these kinases at the interior face of the endocytic compartments, enabling their degradation via the newly entered whole organism and/or OMV, both harboring gingipain activity. It has recently been demonstrated that *Vibrio cholera* OMV enter nonphagocytic host cells and their contents directly interact with NOD1 and NOD2 [96]. A second scenario is that non-vessicle bound gingipains cross the plasma membrane independently, as occurs with RgpA in HeLa cells by a yet to be determined mechanism [97], and directly interact with pools of strictly cytosolic PRR. Three dimensional modeling revealed high similarity between S2 and S3 binding pockets of RgpB and Kgp; however, differences in the S1 pocket architecture ensure distinct substrate specificity, suggesting potentially critical interactions between RIPK1 or RIPK2 and Kgp, likely representing hydrophobic amino groups at the P2 position [98].

Increasing evidence suggests that the kinase domain of RIPK2 maintains protein stability. The reduction of RIPK2 protein levels has been reported in studies that disrupted the catalytic domain of the RIPK2 kinase domain. Inactivation of the ATP binding site of RIPK2 through genetic disruption of key amino acid residues (K38, K47, D164) or by chemical inhibition decreased full-length RIPK2 levels, decreased autophosphorylation and prevented RIPK2-mediated cellular activation or apoptosis [1], [13]. Windheim and coworkers note that the chemical inhibition of RIPK2 activity with SB 203580 decreased RIPK2 levels within 30–60 min, and was not inhibited by MG132 proteasome inhibition or caspase 1 inhibition in preliminary studies. It is possible that the mechanism(s) involved in reduction of RIPK2 in response to disruption of kinase activity or by *P. gingivalis* are not equivalent. However, we may envision a joint scenario where Kgp activity initiates the *P. gingivalis*-induced proteolysis of RIPK2 by cleaving a key lysine in the catalytic region of the kinase domain of RIPK2 (of which there are 12), followed by a host-mediated mechanism(s) to remove the unstable protein. Given the regulation of RIPK1 and RIPK2 by ubiquitination and deubiquitination to modulate cell fate [8], [19], [26], [99], [100], it would be of value to identify the amino acid site(s) of Kgp-induced cleavage of RIPK1 and RIPK2 and determine if they are the lysines involved in kinase activity or targets of ubiquitination. It may not be surprising that *P. gingivalis* evolved with a cysteine protease that targets lysine residues, as these unique amino acids are subject to post-translational modifications, including phosphorylation, ubiquitination and acetylation, to alter protein function. This may provide a mechanism for *P. gingivalis* to dysregulate proteins that are subject to post-translational modification and play a role in host defense.

**Figure 11:**
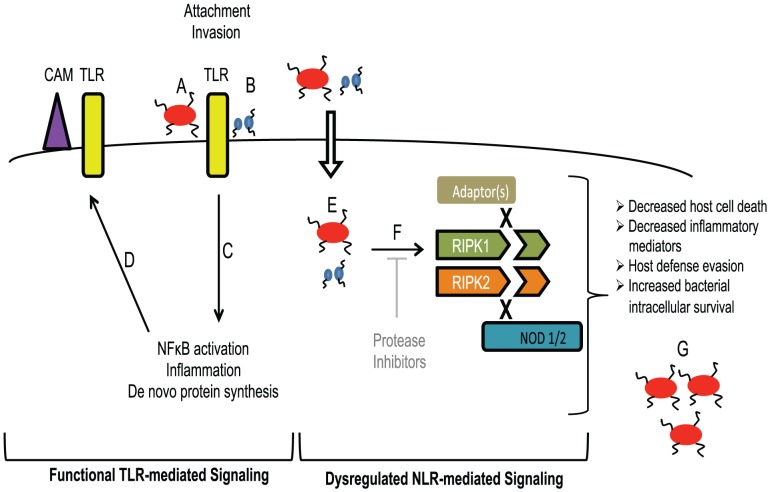
Model of *P. gingivalis* innate immune activation/invasion in endothelial cells. Outcome of innate immune responses is representative as a balance of functional, intact pathways (TLR-left panel) and dysregulated pathways (NLR-right panel). *P. gingivalis* represents a human pathogen that utilizes fimbriae for attachment and invasion of endothelial cells. Fimbriae are not only expressed on whole bacteria (**A**), but within outer membrane vesicles (OMV) that are released from the cell (**B**), as occurs with all Gram-negative bacteria identified to date. Fimbriae bind to TLR2 and MD2 to activate TLR2 and TLR4, resulting in the induction of NF-κB, leading to inflammation, and *de novo* protein synthesis (**C**) of cell adhesion molecules (**CAM**) and TLR (**TLR**) expressed at the cell surface (**D**). Many studies have demonstrated invasion of endothelial cells by *P. gingivalis* (**E**). However, recent studies have shown that OMV (containing fimbriae and active gingipain activity) gain entry into host cells rapidly in a gingipain-dependent manner (**E**), independent of whole organism. Upon entry, intracellular gingipain activity degrades RIPK1 and RIPK2 (abrogated by protease inhibitors) (**F**), resulting in a variety of possible consequences as a function of selective targeting of intracellular NOD1 or NOD2 (NOD1/2) signaling pathways and/or disruption of RIPK1 or RIPK2-mediated cell signaling. Alteration of immune signaling responses may result in decreased host cell death, decreased inflammatory mediator expression, and subsequent enhancement of intracellular bacterial cell survival, all of which contributes to an intracellular niche for *P. gingivalis* (**G**).

The interactions between bacteria with host defense mechanisms are dynamic cellular processes involving pathogen-mediated factors and host signaling cascades, microenvironments and appropriate immune responses. We and others have demonstrated the role of membrane-bound TLR in the recognition and host defense against *P. gingivalis*. However, the roles of intracellular PRR in *P. gingivalis* infection and persistence are poorly understood. Our results demonstrate that *P. gingivalis* Kgp activity induces the proteolysis of key signaling proteins involved in TNF-mediated cell death and NOD-mediated host defense pathways. Further studies will be required to determine the functional consequence of these observations, in a manner that can distinguish between RIPK1 and RIPK2-mediated processes. In addition, studies will examine the internalization of OMV and the extent of gingipain colocalization with PRR, RIPK1, and RIPK2. We propose that the disruption of intracellular signaling facilitates the persistence of *P. gingivalis* in endothelial cells by evading host defense mechanisms and altering cell death pathways (**Figure 11**).

## Materials and Methods

### Ethics statement

This study was carried out in strict accordance with the recommendations in the Guide for the Care and Use of Laboratory Animals of the National Institutes of Health (The Guide). The protocol was approved by Boston University's Institutional Animal Care and Use Committee (IACUC) Protocol Number AN14348. Boston University is committed to observing Federal policies and regulations and Association for Assessment and Accreditation of Laboratory Animal Care (AAALAC) International standards and guidelines for humane care and use of animals. Federal guidelines, the Animal Welfare Act (AWA) and The Guide were followed when carrying out these experiments. Procedures involved euthanasia and harvesting of bone marrow cells. All efforts were made to minimize discomfort, pain and distress.

### Cell culture

Human aortic endothelial cells (HAEC) (Lonza Walkersville, Inc.) or pooled human umbilical vein endothelial cells (HUVEC) (Lonza Walkersville, Inc.) were maintained with Endothelial Growth Medium 2 (EGM-2) (Lonza Walkersville, Inc.) in a 37°C incubator with 5% CO_2_. Experiments were performed with cells (passage 3–8) seeded in 6-well plates at a density of 2–3×10^5^ cells/well in EGM-2 without supplementation of antibiotics.

### Mice

Wild type C57BL/6, *casp2^−/−^*, *casp3^−/−^*, and *casp7^−/−^*, mice were obtained from The Jackson Laboratories and *casp1^−/−^* mice were generously provided by Dr. Robin Ingalls (Boston University School of Medicine). Mice were sacrificed to obtain primary bone marrow derived cells that were differentiated into macrophages with 1X RPMI (cellgro) supplemented with 20% L929-conditioned medium, 10% FBS, penicillin and streptomycin for 7 d. Experiments were performed in 6-well plates at a BMDM cell density of 1.5×10^6^ cells per well in 1X RPMI supplemented with 10% FBS.

### Bacterial strains and growth media

Strains of *Porphyromonas gingivalis* used in these studies are included in **Table S1**. Strains were maintained at 37°C on 5% anaerobic blood agar plates in a GasPak EZ gas generating container system (Becton, Dickinson and Company (BD)). Wild type *P. gingivalis* 381 was used in most of our studies. To evaluate the role of gingipains, isogenic mutant strains of *P. gingivalis* ATCC 33277 were used, including YPP1 (*rgpA^−^*), YPP2 (*kgp^−^*) [57] and RgpA/B (*rgpA^−^* and *rgpB^−^* mutant) [101]. Laboratory-prepared blood agar plates were used in experiments comparing different strains; otherwise, wild type strain 381 was grown on commercial anaerobic blood agar (ABA) plates (BD). Strains YPP1, YPP2 and RgpA/B were grown on plates supplemented with 5 µg/ml erythromycin (Sigma). To evaluate the role of the major fimbriae, we used DPG3 [102], a major fimbriae (*fimA*)-deficient isogenic mutant strain of *P. gingivalis* 381 that has been described as noninvasive/invasion-deficient of endothelial cells *in vitro* [35], [47]. For stimulation assays, organisms were transferred from plates to liquid cultures of bovine heart infusion (BD) broth pH 7.4 supplemented with 5 mg/ml yeast extract (BD), 10 µg/ml hemin (Sigma), 1 µg/ml menadione (Sigma) and antibiotics as described above when necessary and incubated at 37°C in an anaerobic chamber with 85% N_2_, 5% H_2_ and 10% CO_2_. Culture purity was checked by Gram staining.

### Preparation of *P. gingivalis* for co-culture assays

Overnight bacteria cultures were washed three times with phosphate buffered saline (PBS) (cellgro) and resuspended in cell culture medium to achieve a desired working concentration of 1×10^9^ cfu/ml bacteria as previously described [38]. Heat-killed preparations (HK) of *P. gingivalis* were prepared by incubating aliquots of the washed suspension at 60°C for 60 min as previously described [39] or at 80°C for 20 min. Organisms were plated on ABA plates and incubated in an anaerobic chamber for at least 5 d to confirm the concentration of live organism or viability of HK organism. Inhibition of *P. gingivalis* gingipain activity was performed with the previously described [54] arginine-specific gingipain inhibitor carbobenzoxy-Lys-Arg-CO-Lys-N-(CH_3_)_2_ (KYT-1) (Pepta Nova/Peptides International), the lysine-specific gingipain inhibitor carbobenzoxy-Glu(NHN(CH_3_)Ph)-Lys-CO-NHCH_2_Ph (KYT-36) (Pepta Nova/Peptides International) and the general gingipain inhibitor Na-*p*-tosyl-L-lysine chloromethyl ketone hydrochloride (TLCK) (Acros Organics). Washed bacterial suspensions were pre-incubated in 10 µM KYT-1, 10 µM KYT-36, 10 µM KYT-1 and 10 µM KYT36, or 1 mM TLCK for 45 min at 37°C prior to co-culturing with HAEC.

### Arg-X-specific and Lys-X-specific whole cell protease assays

The arginine-X-specific and lysine-X-specific protease activity of *P. gingivalis* was determined using the enzymatic substrate hydrolysis of Nα-benzoyl-DL-arginine 4 *p*-nitroanilide (BAPNA) (Sigma) or N-Tosylglycyl-L-prolyl-L-lysine 4-nitroanilide (Sigma), respectively. *P. gingivalis* preparations were preincubated in 200 mM Tris/HCl buffer, pH 7.6, containing 100 mM NaCl, 5 mM CaCl_2_, and 10 mM cysteine, for 10 min at RT and assayed for amidase activity with 0.5 mM substrate. Formation of *p*-nitroanilide was monitored spectrophotometrically at 405 nm for 30 min using a Biotek Synergy HT plate reader. Each analysis was performed in triplicate.

### Caspase-3 protease activity assay

To determine if specific gingipain inhibitors affected host caspase activity, caspase-3 activity was measured using EnzChek caspase assay kit (Molecular Probes). HUVEC were seeded at 5×10^5^ cells/well in 6-well plates. Cells were untreated or treated with 2 µM staurosporine for 5 h in order to induce caspase-3 activity. Whole cell lysates were collected and placed in reaction buffer (10 mM PIPES, pH 7.4, 2 mM EDTA, 0.1% CHAPS) with dimethyl sulfoxide (DMSO) (Sigma), KYT-36 (3 µM), or Ac-DEVD-CHO caspase inhibitor (20 µM) and incubated for 10 min. Lysates were placed into 96-well plates containing Z-DEVD-AMC substrate (100 µM). Fluorescence (excitation/emission ∼342/441 nm) was measured after 60 min. The fold change in fluorescence was calculated relative to untreated. Data represents the average of three replicate experiments.

### Co-culture/Stimulation assays

Cell stimulation assays were performed in a 37°C humidified chamber with 5% CO_2_ and were performed at least three times. Cells were treated with medium (new cell culture medium), live organisms, inactivated organisms or with commercially available synthetic or purified cell agonists at a concentration and incubation period indicated in each figure legend. Agonists used in this work include synthetic bacterial tripalmitoylated lipopeptide Pam_3_CSK4 (TLR1/TLR2 agonist), synthetic diacylated lipoprotein of *Mycoplasma fermentans* FSL-1 (TLR2/TLR6 agonist), ultrapure *P. gingivalis* strain ATCC 33277 LPS (TLR2/TLR4 agonist), ultrapure *E. coli* strain 0111:B4 LPS (TLR4 agonist), γ-D-glutamyl-meso-diaminopimelic acid (iE-DAP, NOD1 agonist), N-acetylmuramyl-L-alanyl-D-isoglutamine (MDP, NOD2 agonist), acylated iE-DAP (C12-iE-DAP, NOD1 agonist) acylated MDP (L18-MDP, NOD2 agonist) control peptides for iE-DAP or MDP including iE-Lys (γ-D-glutamyl-lysine, a dipeptide present in the peptidoglycan of Gram-positive bacteria) or with an inactive D-D isomer of MDP [103] (all from InvivoGen), and recombinant human TNF (TNF-R1 agonist) (BD Pharmingen).

### Inhibition of endothelial cell protein synthesis or caspase activity

For some experiments, endothelial cells were pretreated with cellular inhibitors including the general protein synthesis inhibitor cycloheximide (Sigma), caspase inhibitors zVAD-FMK (R&D Systems) or Boc-D-FMK (BioVison), or with vehicle control, DMSO.

### Apoptosis assays and flow cytometry

Induction of apoptosis of endothelial cells was performed with treatment with staurosporine, or co-treatment with cycloheximide and recombinant TNF at the concentration and the time points indicated. Cells were either analyzed by Western blot to monitor caspase 3 cleavage or by flow cytometry to monitor Annexin V and propidium iodide (PI) staining. For flow cytometry, adherent cells were removed with a trypsin-EDTA solution and pooled with the floating fraction. Cells were stained with Annexin V eFluor 450 Apoptosis Detection Kit (eBioscience) using the manufacturer's protocol. Just before acquisition, PI was added to distinguish dead versus apoptotic cells. At least 10,000 events were acquired on a BD LSR II flow cytometer. Data were analyzed using FlowJo (Tree Star, Inc.).

### Sample processing and Western blot analysis

Stimulation assays were ceased by immediately placing cell culture plates on ice washing cells three times with ice-cold PBS and addition of RIPA lysis Buffer [20 mM Tris HCl, pH 7.5, 150 mM NaCl, 1% NP-40, 1 mM EDTA, 1 mM EGTA, 1% sodium deoxycholate, 2.5 mM sodium pyrophosphate, 1 mM β-glycerophosphate] supplemented with complete protease inhibitor cocktail (Roche) and PhosSTOP phosphatase inhibitor cocktail (Roche). Cells were scraped from the plate, cell lysates were harvested and placed on ice for 30 min with occasional vortexing. Samples were clarified of cellular debris by micro centrifugation at 13,200 rpm for 15 min at 4°C. The protein concentration of the clarified lysates was determined by a bicinchoninic acid protein assay (Sigma). Samples were prepared for Western blot by combining clarified whole cell lysates with Laemmli sample buffer (Bio-Rad Laboratories) supplemented with dithiothreitol (Sigma) for a final concentration of 100 mM, and heated for 10 min at 100°C to denature proteins. Samples (20–40 µg) were separated on SDS-PAGE gels, transferred to polyvinyldifluoride membranes, blocked in 5% milk and immunoblotted for target proteins.

Primary antibodies used in this study include a a mouse monoclonal antibody to caspase 3 (clone 3G2), as rabbit polyclonal antibody to cleaved caspase 3 (clone Asp175), a rabbit polyclonal antibody to NOD1 (Cell Signaling Technology), a mouse monoclonal antibody to NOD2 (Biolegend), rabbit antibody to PARP (Cell Signaling Technology), a mouse antibody to RIPK1 (clone G322-2) (BD Biosciences), a rabbit polyclonal antibody to the N′-terminus of RIPK2 (Axxora Platform), a mouse monoclonal antibody to the C′-terminus of RIPK2 (Abcam), and a rabbit monoclonal antibody to GAPDH (clone 14C10; Cell Signaling Technology) as a sample loading control. Secondary antibodies include HRP-linked anti-mouse IgG, (GE Amersham) and HRP-linked anti-rabbit IgG (Cell Signaling Technology). Membranes were exposed to enhanced chemiluminescence (GE Amersham) and signal intensity was documented by exposing membranes to film. All Western blots performed to monitor the levels of RIPK2 in this study were analyzed with both N′-terminal- and C′-terminal-directed antibodies. However, all blots for RIPK2 detection presented in this report are with the N′-terminal antibody, except for Figure 1B that includes both N′-terminal and C′-terminal antibody immunoblots.

### Densitometry

Quantification of Western Blot protein signal intensity levels was acquired by either a charged coupled device camera, LAS 4000 luminescent image analyzer (Fujifilm) and assessed by Multigauge 3.0 software (Fujifilm) or by a Kodak DC290 zoom digital camera and assessed by Kodak 1D v.3.6.0 software. Membrane background values were subtracted from protein intensity values. Data is presented as the mean and standard error of the mean (SEM) ratio of the target protein (NOD1, NOD2, RIPK1, or RIPK2) to the loading control (GAPDH).

### Cell-free assays

Washed suspensions of overnight cultures of *P. gingivalis* 381 were prepared similarly as above, except suspensions were resuspended in 30 mM HEPES buffer (Lonza Walkersville, Inc.). *P. gingivalis* strain 381 was pretreated with 10 µM KYT-1, 10 µM KYT-36, 10 µM KYT-1 and 10 µM KYT-36, 1 mM TLCK, 100 µM zVAD-fmk, 100 µM BocD-fmk with or vehicle controls (HEPES, DMSO or acid water) for 45 min, then immediately co-cultured with 0.1 µg recombinant RIPK2 kinase (Invitrogen) for 1 h in a humidified chamber at 37°C with 5% CO_2_. Ten percent of initial reactions were harvested for gel loading controls. Reactions were stopped by the addition of SDS-PAGE loading dye and analyzed by Western blot analysis with an antibody to the N′-terminal kinase domain of RIPK2.

### ELISA

Cell culture supernatants were collected from stimulation assays (performed in triplicate), clarified of bacteria and cellular debris and assayed in triplicate by ELISA. Supernatants from HAEC were assayed with ELISA kit specific for detection of human IL-8 according to the manufacturer's instructions with limit of detection of 3.1 pg/ml (BD Biosciences).

### Statistics

Statistical analysis of ELISA data, densitometry and caspase 3 assay activity data was performed with GraphPad Prism 5 software for two-tailed unpaired T test (α = 0.05).

## Supporting Information

Figure S1 **Schematic of antibody recognition sites of RIPK1 and RIPK2 antibodies used in this study.** Antibodies used include **A**) a carboxyl (C′)-terminal-targeted antibody that detects the intermediate region and death domain of RIPK1, and **B**) an amino (N′)-terminal polyclonal antibody that detects the kinase domain of RIPK2 (AA 11–30) and a C′-terminal monoclonal antibody that detects the CARD of RIPK2 (AA 431–541).
(EPS)

Figure S2 **Proteolysis of RIPK1 and RIPK2 in HUVEC by** ***P. gingivalis*** **DPG3 is attenuated.** HUVEC were treated with medium (M,-), *P. gingivalis* 381 at a MOI of 10 (**10**) or 100 (**100**), or with the *fimA* mutant, DPG3, at a MOI of 10 or 100 for 6 h. Whole cell lysates were analyzed for the detection of RIPK1 (left panel) or RIPK2 (right panel). Full-length RIPK2 is indicated with an arrow. Prominent *P. gingivalis*-induced LMW bands are indicated with asterisks. MW ladder is indicated on the left in kDa. GAPDH was detected as a loading control. Densitometric analysis is presented below respective blots as the percent mean ratio of RIPK1 (or RIPK2) to GAPDH protein levels (arbitrary densitometric units (A.D.U.).
(EPS)

Figure S3 **The** ***P. gingivalis*** **-induced LMW bands in HUVEC are not new protein products.** HUVEC were untreated (−) or pretreated (+) with 25 µg/ml cyclohexamide (**CHX**) for 30 min, followed by treatment with medium (**M**), 50 ng/ml TNF (**TNF**) or *P. gingivalis* strain 381 (**381**, MOI 100) for 2 h. Whole cell lysates were analyzed by Western blot for the detection of RIPK1 (left panel) RIPK2 (right panel). Prominent *P. gingivalis*-induced LMW bands are indicated with asterisks. MW ladder is indicated on the left in kDa. GAPDH was detected as a loading control. Cleaved and full-length caspase 3, and PARP were monitored to confirm the effect of CHX.
(EPS)

Figure S4 **TLR and NLR agonist-induction of HAEC IL-8 levels.** HAEC were treated with medium, 10 µg/ml Pam_3_CSK4, 10 µg/ml FSL-1, 10 µg/ml *P. gingivalis* 381 LPS, 1.0 µg/ml *E. coli* 0111:B4 LPS, 100 µg/ml iE-DAP, 1000 ng/ml C12-iE-DAP, 100 µg/ml MDP, 1000 ng/ml L18-MDP or 100 ng/ml recombinant human TNFα for **A**) 6 h, **B**) 12 h and **C**) 24 h and evaluated for cell culture supernatant IL-8 levels. Statistical significance between groups was performed by un-paired, two-tailed T-test (α = 0.05), and indicated with asterisk, with * = *p*<0.05, ** = *p*<0.01 and *** = *p*<0.001, or ns = non significant.
(EPS)

Figure S5 **Induction of apoptotic markers in HUVEC in response to classical apoptotic stimuli or** ***P. gingivalis*** **.** HUVEC were treated with medium, *P. gingivalis* 381 (MOI 10 or 100), 2 µM staurosporine (STS) 25 µg/ml cycloheximide (CHX), 10 ng/ml TNFα, or co-treated with 25 µg/ml CHX and 10 ng/ml TNFα for 6 h. **A**) Flow cytometric analysis of annexin V and propidium iodide (PI) staining of HUVEC treated as indicated. Apoptotic cells which retain membrane integrity are Annexin V positive/PI negative, whereas necrotic cells with compromised membrane integrity are Annexin V positive/PI positive. **B**) Whole cell lysates were analyzed for the detection of full-length (fl.) and cleaved (c.) caspase 3 (with an antibody that detects a 17- and 19-KDa fragment) and full-length (fl.) and cleaved (c.) PARP. MW ladder is indicated on the left in kDa. GAPDH was detected as a loading control.
(EPS)

Figure S6 **Boc-D-FMK has minimal effect in** ***P. gingivalis*** **-induced proteolysis of RIPK1, RIPK2 and PARP.** HUVEC were pre-treated (**Pre-Tx**) with medium (**M**), 0.25% DMSO vehicle control (**C**), or 100 µM Boc-D-FMK (**Boc**) for 1 h. HUVEC were then treated with medium (**M**), *P. gingivalis* 381 (MOI 100) or 2 µM staurosporine (**STS**) for 1.5 h. Whole cell lysates were analyzed for RIPK1 (top panel) RIPK2 (mid panel), PARP (lower panel) and GAPDH (bottom panel). Full-length RIPK1 and RIPK2 are indicated with arrows. Prominent *P. gingivalis*-induced LMW bands are indicated with asterisks. MW ladder is indicated on the left in kDa.
(EPS)

Figure S7 **Lysine-specific gingipain (Kgp) activity mediates** ***P. gingivalis*** **-induced modification of PARP in HAEC.** **A**) Kinetics of PARP levels in HAEC in response to *P. gingivalis* 381. HAEC were treated with medium (**M**) or with *P. gingivalis* strain 381 (MO1 100) for 0.25, 0.5, 1, 2, 6, 12, 24 or 48 h. Whole cell lysates were analyzed for the detection of PARP (116-kDa). Full-length PARP is indicated with an arrow. MW ladder is indicated on the left in kDa. GAPDH was detected as a loading control. **B**) *P. gingivalis* strain 381 was pretreated with 10 µM KYT-1, 10 µM KYT-36, 10 µM KYT-1 and 10 µM KYT-36, 1 mM TLCK, or vehicle controls (DMSO or acid water) for 45 min. HAEC were then immediately co-cultured with medium, or with the pretreated preparations of *P. gingivalis* 381 (MOI 100) for 2 h. Whole cell lysates were analyzed for PARP and GAPDH. **C**) HAEC were treated with medium or *P. gingivalis* strain 381, strain ATCC 33277, or isogenic mutants of 33277: YPP1 (*rgpA^−^*), or with YPP2 (*kgp^−^*) at an MOI of 100 for 2 h. Whole cell lysates were analyzed for PARP and GAPDH.
(EPS)

Table S1 **Bacterial strains used in this study.**
(DOC)
