# Supplementary material for: Pathogen-Mediated Proteolysis of the Cell Death Regulator RIPK1 and the Host Defense Modulator RIPK2 in Human Aortic Endothelial Cells
Source: PLoS Pathog. 2012 Jun 7;8(6):e1002723. doi: 10.1371/journal.ppat.1002723 (PMC3369954; doi:10.1371/journal.ppat.1002723)
Supplement: Table S1 — Bacterial strains used in this study. (DOC) [file ppat.1002723.s008.doc]

| ***P. gingivalis* strain** | **Genotype or relevant properties** | **Rgp-X**  **Activity***  **(mOD/min)** | **Kgp-X Activity***  **(mOD/min)** | **Source**  **[reference]** |
| --- | --- | --- | --- | --- |
| 381 | Wild type | 364.3 ± 2.7 | 87.0 ± 1.4 | Laboratory Collection |
| DPG3 | *FimA*-deficient isogenic mutant strain of 381 | 371.5 ± 1.2 | 92.5 ± 1.6 | [10] |
| ATCC 33277 | Wild type | 426.8 ± 4.9 | 88.8 ± 0.1 | Laboratory Collection |
| YPP1 | *rgpA*-deficient isogenic mutant of strain 33277 | 257.1 ± 0.3 | 81.3 ± 1.5 | [57] |
| YPP2 | *kgpA*-deficient isogenic mutant of strain 33277 | 413.9 ± 3.7 | 7.3 ± 0.2 | [57] |
| RgpA/B | *rgpA-* and *rgpB*-deficient isogenic mutant of strain 33277 | 0.5 ± 0.3 | 100.0 ± 1.6 | [101] |

*Rgp-X and Kpg-X activity were assessed as described in the Methods and Methods.
